# Dyslipidemia and Cardiovascular Disease Risk Factor Management in HIV-1-Infected Subjects Treated with HAART in the Spanish VACH Cohort

**DOI:** 10.2174/1874613600802010026

**Published:** 2008-03-24

**Authors:** Pere Domingo, Ignacio Suarez-Lozano, Ramón Teira, Fernando Lozano, Alberto Terrón, Pompeyo Viciana, Juan González, Mª José Galindo, Paloma Geijo, Antonio Vergara, Jaime Cosín, Esteban Ribera, Bernardino Roca, Mª Luisa Garcia-Alcalde, Trinitario Sánchez, Ferran Torres, Juan Ramón Lacalle, Myriam Garrido

**Affiliations:** 1 Hospital de la Santa Creu i Sant Pau; 2 H. I. Elena–Huelva; 3 H. de Basurto-Bilbao; 4 H. de Valme-Sevilla; 5 H. Clinico Puerto Real; 6 H. V. del Rocío-Sevilla; 7 H. La Paz-Madrid; 8 H. Clinico-Valencia; 9 H. V de la Luz-Cuenca; 10 H. SAS-Jerez; 11 H. Gregorio Marañon-Madrid; 12 H. Vall D´hebron-Barcelona; 13 H. General de Castellon; 14 H. de Cabueñes-Asturias; 15 H. V. del Rosell-Cartagena; 16 Dpto. de Estadística, Facultad de Medicina, Universitat Autònoma de Barcelona; 17 Dpto. Estadística, Facultad de Medicina Universidad Sevilla; 18 Data Management VACH Group

**Keywords:** HIV-1 infection, antiretroviral therapy, HIV-1 protease inhibitors, non nucleoside reverse transcriptase inhibitors, dyslipidemia, cardiovascular risk, coronary heart disease, VACH

## Abstract

**Background:**

There is increasing evidence that metabolic adverse effects associated with antiretroviral therapy may translate into an increased cardiovascular risk in HIV-1-infected patients.

**Objectives:**

To determine the prevalence of risk factors for cardiovascular disease (CVD) among HIV-1-infected persons, and to investigate any association between them, stage of HIV-1 disease, and use of antiretroviral therapies.

**Methods:**

Multicentric, cross-sectional analysis of CVD risk factors of treated patients in the VACH cohort. The data collected includes: demographic variables, cigarette smoking, diabetes mellitus, hypertension, dyslipidemia, body mass index, stage of HIV-1 infection, and antiretroviral therapy.

**Results:**

The analysis included 2358 patients. More than 18% of the study population was at an age of appreciable risk of CVD. 1.7% had previous CVD and 59.2% were smokers. Increased prevalence of elevated total cholesterol was observed among subjects receiving an NNRTI but no PI [odds ratio (OR), 3.34; 95% confidence interval (CI), 1.77–6.31], PI but no NNRTI (OR, 4.04; 95% CI, 2.12–7.71), or NNRTI + PI (OR, 17.77; 95% CI, 7.24–43.59) compared to patients treated only with nucleoside reverse transcriptase inhibitors (NRTI). Higher CD4 cell count, lower plasma HIV-1 RNA levels, clinical signs of lipodystrophy, longer exposure times to NNRTI and PI, and older age were all also associated with elevated cholesterol levels. The use of lipid lowering agents was very low among our patients.

**Conclusion:**

Patients in the VACH cohort present multiple known risk factors for CVD, and a very low rate of lipid lowering therapy use. NNRTI and/or PI-based antiretroviral therapies are associated with the worst lipid profile. This is more frequent in older subjects with greater CD4 counts and controlled HIV-1 replication.

## INTRODUCTION

The widespread use of highly active antiretroviral therapy (HAART) has converted HIV-1 infection into a chronic manageable illness that needs lifelong therapy. However, HAART is associated with metabolic side effects including hypercholesterolemia, hypertriglyceridemia, insulin resistance and more rarely diabetes mellitus, and possibly arterial hypertension [1-4]. HAART-associated dyslipidemia is associated with accelerated atherosclerosis [5-7] and signs of endothelial dysfunction [8]. How all these facts are

translated into clinical events of cardiovascular disease on a population level has been examined by a number of studies including cohort studies from the French Hospital Database [9], the HOPS Cohort [10], and the D:A:D multicohort study [11-13]. Overall, data from these cohorts suggest an increased risk of coronary heart disease (CHD) for HIV-1-infected patients on HAART [9-3].

To assess the risk of treatment-associated cardiovascular risk factors, we performed a cross-sectional analysis of the HIV-1-infected patients included in the Spanish VACH Cohort. The objectives of the present analysis were to determine the proportion of patients with an increased risk profile for cardiovascular disease. Furthermore, we tried to identify factors associated with the increased risk profile. Addition ally, our research was focused on the management of dyslipidemia in our cohort.

## PATIENTS AND METHODS

### Design

The study is a multi-centre, cross-sectional study of the prevalence and management of dyslipidemia and other established CHD/CVD risk factors in all HIV-1-infected subjects, aged 18 or above, treated with HAART, in routine clinical practice from the Spanish VACH cohort treatment centers. Characteristics of VACH Cohort have been described elsewhere [14].

### Study Population

Male or female subjects aged 18 years or above at the time of enrollment with a documented HIV-1 infection, which attend VACH cohort outpatient HIV-1 treatment centers for routine, scheduled, clinical appointments, were eligible for this study.

In order to be eligible, subjects must have been on at least three antiretroviral drugs, at the time of the study visit. Antiretroviral (ARV) naïve subjects or ARV experienced, but currently untreated subjects or those currently treated with NRTI bi- or mono therapy were not eligible for this study. However, for the purpose of the study, we included patients treated with 3 NRTIs. Subjects who were hospitalized or have a frank cognitive impairment such as delirium or dementia on enrolment were not eligible either. Informed consent was obtained from the patients at study entry

### Data Collection

In the VACH Cohort, data are prospectively collected according to standardized criteria, and are electronically stored in the Aplicación de Control Hospitalario (AC&amp;HTM), an application specifically developed for the management of the cohort data. On enrolment, standardized data collection electronic forms were completed at the sites providing information from physical examination, patient interview and patient case notes, concerning family history of coronary heart disease, patients’ prior history of CVD and diabetes, cigarette smoking, blood pressure, therapy for diabetes mellitus, lipid-lowering and anti-hypertensive therapy, the presence of clinical signs of lipodystrophy and fasting serum lipid levels. Further, all cumulative data characterizing the patient’s underlying HIV-1 infection since inclusion in any of the individual cohorts were collected, including information on demography, antiretroviral therapy, CD4 cell counts and HIV-1 viral loads. Dates of diagnosis of all AIDS-defining diseases are recorded, using the 1993 clinical definition of AIDS from the Centers for Disease Control and Prevention [15].

## VARIABLES

### HIV-1 Laboratory Parameters

CD4 cell count was stratified in strata of 100 x 10^6^ (cells/l) or assessed as a continuous variable (log_2_ transformed). Similarly, HIV-1 RNA was stratified in strata of: <  500, 501–10 000, 10 001–100 000, and > 100 000 copies/ml, and also assessed as a continuous variable (log_10_ transformed).

### Antiretroviral Therapy

Four categories were predefined: (i) currently receiving only NRTI; (ii) currently receiving NNRTI and NRTI but not PI; (iii) currently receiving PI and NRTI but not NNRTI; or (iv) currently receiving PI, NNRTI and NRTI. Previous antiretroviral therapy exposure was modelled as cumulative time spent using each of the three drug classes.

### Cardiovascular Risk Factors and Managing Therapies

The grouping of the risk factors assessed was defined prior to the initiation of the analysis. CVD risk factors were assessed as dichotomous categorical variables, where the cut-off levels chosen were conservative estimates of ‘high risk’ based on levels used for risk scoring in the background population [16-19]. Patients were considered to be at high risk for CVD if they had dad a prior CVD event or had diabetes or more than two risk factors accounting for a CVD 10 yr risk > 20%. Moderate-high risk was considered if patients had more than two risks factors accounting for a CVD 10 yr risk of 10-20%, whereas moderate risk was considered when patients more than 2 risk factors and a CVD 10 yr risk of less than 10%. Finally low risk patients were those who had no or only a risk factor.

### Cardiovascular Risk Calculation

Ten-year CHD risk estimates were calculated in accordance with the equations of Wilson *et al*. [16] with use of sex-specific risk calculations based on age, total and HDL cholesterol levels, systolic and diastolic blood pressure, presence of diabetes (defined as a fasting glucose level of 140 mg/dL), family history of CHD, personal history of CHD, and smoking status. The equation estimates the 10-year risk for CHD events, including angina pectoris, myocardial infarction, and death due to CHD.

### Statistical Analyses

We used frequencies, percentages and its 95% CI to describe categorical data, and median with interquartile range (IQR) for continuous variables. Univariable analysis was performed by means of the chi-squared and the Kruskal-Wallis tests to compare categorical and continuous baseline demographic, clinical and laboratory characteristics. Association of CVD risk factors with antiretroviral therapy, demographic, clinical and laboratory parameters were tested in univariable logistic regression models. Multivariable logistic regression was then performed to identify parameters independently associated with the presence of CVD risk factors. The multivariable model included all parameters significantly associated with the risk factor assessed, at a level of p< 0.05 in the univariable model.

The analysis was performed using SAS version 9.1.3 software (SAS Institute Inc, Cary, North Carolina, USA) and the level of significance was established at the 0.05 level (two-sided).

## RESULTS

### Demographics

By April 2004, the central database contained information on 2358 patients enrolled in VACH from fifteen participating Hospital cohorts. The patient characteristics are shown in Table **1**.

### Antiretroviral Therapy

On enrolment, 9.9% were receiving a regimen containing NRTI only, 46.4% were receiving NNRTI-based therapy, 41.9% PI-based therapy and 1.7% was on a regimen containing all three drug classes (Table **1**). Sixty four percent of patients taking PI-based therapy had ritonavir-boosted PI on board while 0.02% were taking atazanavir. Overall, 72.6% of

the study population had at any time been exposed to PI with a median exposure time of 3.3 years (IQR, 0–5.8 years), 67.0% had been exposed to NNRTI with a median exposure time of 1.5 years (IQR, 0–3.6 years) and 99.9% had been exposed to NRTI with a median exposure of 4.3 years (IQR, 2.0–6.2 years) (Table **1**).

### CVD Risk Factors and Association with Antiretroviral Therapy

CVD risk factors were prevalent in the study population (Table **2**). More than 18% of the study population was in an age group constituting a CVD risk factor. Thirteen percent had a family history of coronary heart disease, and 1.7% had a previous history of CVD. Almost 60% of the study population was current cigarette smokers.

### Serum Total Cholesterol

**Fig. (1):**
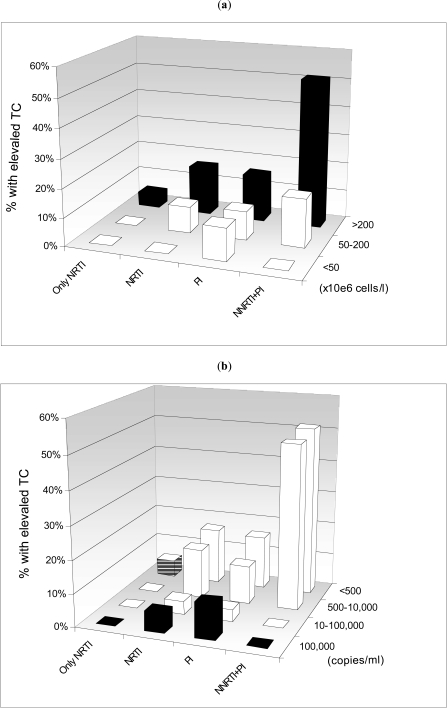
Prevalence of elevated total cholesterol (> 6.2 mmol/l) according to current antiretroviral therapy (ART), CD4 cell count **(b)** (a) and HIV RNA **(b)** at baseline. The four ART categories are: NRTI (currently receiving only NRTI), NNRTI (currently receiving NNRTI and NRTI but not PI), PI (currently receiving PI and NRTI but not NNRTI), and PI/NNRTI (currently receiving PI, NNRTI and NRTI).

The association of antiretroviral therapy with lipid levels is shown in Table **2**. Assessed from median cholesterol levels (Table **2**) and in univariable models (Table **3**), patients currently using regimens containing all three drug classes were at increased risk of having a high total cholesterol when compared with patients using regimen containing only NRTI. This pattern remained unchanged after controlling for other risk factors (Table **4**). In a univariable logistic model for cumulative antiretroviral therapy exposure time, the OR increment of risk for elevated total cholesterol was 9% (p=0.005), 2% (p=0.251) and 2% (p=0.314) per year of exposure to NRTI, NNRTI and PI, respectively (Table **3**). The level of immunodeficiency and plasma HIV-1 RNA were independently associated with elevated total cholesterol after adjustment for other factors (Fig. **1**). Overall, the adjusted risk of having elevated total cholesterol increased by 31% per twofold increase in CD4 cell count [OR, 1.31; IC95%: 1.16-1.47 per log_2_CD4, P <  0.001] (Fig. **1**). In all antiretroviral therapy groups, higher HIV-1 viral load was associated with a decreased risk of elevated total cholesterol (Fig. **1**); overall the adjusted OR was 0.77 (95%CI: 0.68-0.88), P <  0.001, per 1 log_10_ increase in HIV-1 RNA.

### Serum Triglycerides

In a univariable logistic model for cumulative antiretroviral drug exposure time, the OR for elevated triglycerides was 1.05 (95%CI, 1.02-1.09), 1.02 (95%CI, 0.97-1.07) and 1.06 (95%CI, 1.02-1.09) per year of exposure to NRTI, NNRTI and PI, respectively, associations essentially unchanged in the multivariable model (Tables **3** and **4**). Overall, the adjusted risk of elevated triglycerides decreased with increasing HIV-1 RNA [OR (95%CI), 0.86(0.78 to 0.95) per 1 log_10_ increase; P = 0.002], and there was also a significant association with CD4 cell count [OR, 1.20 (IC95%: 1.10 to 1.31) per log_2 _CD4 twofold increase; P <  0.001].

### Serum HDL-Cholesterol

None of the current regimens were associated with an increased risk of low HDL-cholesterol (Tables **2-4**) in the multivariate logistic model. The associations of CD4 cell count and HIV-1 viral load were similar for the absolute value of HDL-cholesterol and for total cholesterol.

### Hypertension

More than 20% of the study population had hypertension. In a univariable logistic model, regimens containing NNRTI, PI or both drug classes were associated with a non statistically different risk of being hypertensive (Tables **3** and **4**) which was explained by a strong correlation of hypertension with other factors (age, sex and BMI).

### Diabetes

The overall prevalence of diabetes was 7.3%. In a univariable model, all regimens were associated with a differen

tial risk of diabetes (Tables **3** and **4**). The adjusted multivariate increment of risk per year of exposure was 8% for NRTI (p=0.011), 10% for NNRTI (p=0.031) and 4% for PI (0.243) (Table **4**).

### Body Composition

In all regimen groups there were few obese patients (Table **[Table T2]**). Antiretroviral therapy was highly associated with the presence of clinical lipodystrophy, with the highest risk among patients receiving a regimen containing all three drug classes (Tables **3** and **4**). When assessed as an explanatory variable, lipodystrophy was associated with the presence of several of the CVD risk factors discussed above (Table **5**). In a multivariable model including the total study population, and adjusting for co-variables as listed in Table **5**, the adjusted OR for the association of lipodystrophy with elevated total cholesterol was 0.90 (0.65-1.23; P = 0.494), elevated triglycerides 1.36 (1.06-1.75; P = 0.017) and decreased HDL 1.19 (0.90.1.58; P = 0.214). The presence of lipodystrophy was associated with an increased risk of hypertension and diabetes OR, 1.47 (95%CI, 1.14–1.90; P = 0.003) and 1.71 (95%CI, 1.17–2.49, P = 0.005), respectively.

### Cardiovascular Risk Assessment

Ten-year CHD risk estimates for HIV-1-infected subjects treated with HAART in the VACH cohort are shown in Table 6. There was no clustering of CHD risk for any treatment group. The 10-year CHD of moderate-high or high risk estimate was significantly increased among HIV-1-infected patients with fat redistribution, compared with that for those with no redistribution: 48.5% versus 37.9%, (P <  0.001) (Table5). Ten-year CHD risk estimates were studied by current HAART regimen (Table 6) and by cumulated ART exposure (Table 4). Predictors of a moderate-high CVD risk estimate obtained from the multivariate logistic model were: OR (95%CI; P value): 0.63 (0.28-1.42; P = 0.266), 1.13 (0.52-2.46; P = 0.756), and 1.91 (0.48-7.57; P =0.359) for NRTI, PI, NNRTI+PI current ART regimens, respectively.

### Lipid-Lowering Therapy (LLT)

The proportion of patients who fulfilled criteria for being treated with LLT were 11.3%, 18.5%, 17.9%, and 26.8%, respectively for patients treated with NRTI only or with NNRTI, PI or NNRTI and PI, respectively (P = 0.0243). However, only between a fourth and a fifth of those patients were currently receiving LLT

## DISCUSSION

In the VACH population we have observed a high prevalence of multiple risk factors for CVD. VACH has the strength of having included more than 2000 treated patients with details concerning CVD risk factors, with an almost insignificant proportion of missing data. Similar to other studies [13], we found that regimens containing drugs from both the PI and NNRTI classes were associated with the highest prevalence of dyslipidemia. Furthermore, we also observed that hypercholesterolemia was associated with a higher CD4 cell count, a lower HIV-1 plasma viral load, the presence of clinical signs of lipodystrophy and older age. These findings, and those of others [13, 20], suggest that immune reconstitution phenomena may play some role in the development of antiretroviral-associated dyslipidemia.

However, our findings have inherent limitations. Firstly, our findings are not applicable to other populations since the diversity of the study population, including women, minorities and means of acquiring HIV-1 infection, implies that the study may not be representative of the HIV-1-infected population in other industrialized countries. Furthermore, the Mediterranean basin is an area of low incidence in terms of coronary heart disease and its risk factors, which may make comparisons with similar studies difficult [21, 22]. However, our study suggests that even in such an environment, HIV-1-infected patients on HAART develop comparable metabolic disturbances to other geographically-based cohorts [23-25]. Since information concerning certain other potential risk factors for CVD, such as diet, physical activity and genetic factors, was not collected in our study, this may restrict the validity of our findings only to populations similar to ours.

Other limitations are related to the observational design of the study. Firstly, the results presented are only associations from which no conclusions regarding causality can or should be drawn. Secondly, due to the observational design of the study, many measurements (blood pressure, lipid levels) are expected not to be always conducted in a uniform manner. However, international and national standardization of serum lipid measurements have been accomplished throughout the guidelines of the NCEP ATP II and the Spanish Society of Hypertension [19, 26]. On the other hand, the relatively low proportion of missing data should be noted (Table **1**), which implies that the prevalence of the individual risk factors is precise.

Dyslipidemia was most strongly correlated with antiretroviral regimens currently being used, and less with a history of previous exposure to the different drug classes. This is consistent with previous reports, in which the PI-associated dyslipidemia occurred shortly after beginning therapy [27, 28] and it is also consistent with studies showing that a switch from PI to NNRTI-based or NRTI-only regimens is associated with attenuation or resolution of dyslipidemia [29, 30]. The average increases in lipid levels [2, 13, 31, 32], comparing levels during PI therapy with either pre-therapy levels or levels in PI-naïve HIV-1-infected patients, were 28% for total cholesterol and 96% for triglycerides. We observed no difference in risk of low HDL-cholesterol among patients treated with PI, NRTI or NNRTIs, data reported by others as well [13, 26]. Duration of PI therapy did not influence the level of HDL cholesterol [32], whereas duration of NRTI was associated with a higher risk of low HDL-cholesterol.

The association between NNRTI-containing regimens and dyslipidemia has rarely been reported [13], although, in phase I studies of efavirenz in HIV-1-uninfected subjects revealed increases in total cholesterol levels of 10–20% in some subjects [3], whereas cohort and trial data suggest that 18-20% of NNRTI-treated patients develop dyslipidemia [23-25, 34]. Although a different lipid profile between the two NNRTIs used has been frequently claimed [30] , no differences were reported in HIV-1-infected individuals [35]. In concordance with our results, an increase in HDL-cholesterol with NNRTI has been reported [36]. Consistent with previous reports, NRTI-only therapy was associated with lower rates of elevated total cholesterol [35, 37].

We found a strong association between elevated total cholesterol level and higher CD4 cell counts, which was present within each treatment category. Nevertheless, within each CD4 cell count stratum, the effect of antiretroviral therapy was clearly observed, which indicates that the effect of antiretroviral substances certainly cannot solely be explained by a reversal to ‘normal’ pre-disease cholesterol levels as a result of improved cellular immunity. The level of HDL-cholesterol, although to a lesser extent, likewise increased with more conserved cellular immunity, consistent with observations in the pre-HAART era [38]. For total cholesterol, the association with HIV-1 viral load was the inverse of the association with CD4 cell count. The latter has also been reported from other studies [39]. However, it could also be possible that these findings merely reflect the cumulative exposure to antiretrovirals.

In the VACH study, we have observed a high prevalence of other known and potential CVD risk factors among patients receiving either PI or NNRTI, including cigarette smoking, diabetes, hypertension and altered body composition. The overall prevalence of diabetes mellitus in the VACH study was 7.3%, similar to other studies that have shown an association between diabetes mellitus and use of PI [1, 27, 31], and recently with NRTI exposure [40]. A few studies have reported an increased prevalence of hypertension in PI-treated patients [3, 4] or in conjunction with lipodystrophy [41]. In our study, the associations between antiretroviral drug regimens and hypertension in univariable logistic models were no longer present after adjustment for other factors associated with hypertension. This is in odds with data from the DAD cohort showing no deleterious effect of any class of antiretrovirals on blood pressure [42]. There was a marked association between dyslipidemia and several of the other CVD risk factors on the one hand and clinical lipodystrophy on the other [43]. Current guidelines for the management of dyslipidemia in HIV-1-infected patients have been developed [44]. Assuming that dyslipidemia in HIV-1-infected will have similar long-term consequences to dyslipidemia in the general population, these guidelines also assume that the benefits of lipid lowering interventions will also extend to HIV-1-infected persons, and thus these are based on CVR stratification while the goals to be achieved are based on target values for LDL cholesterol and non-HDL cholesterol [43]. Despite that, we found that only between a fourth and a fifth of patients in our cohort needing LLT were effectively being treated, a figure somwwhat higher than those reported in previous years [34]. This finding may have many causes, but among them the lack of LLT knowledge by HIV-1 treating physicians might be considered. In a recent report, it was shown that when dyslipidemic HIV-1-infected patients were addressed to a specialized lipid clinic, the percentage of those being treated with LLT significantly increased [45].

Recently, the issue of how young persons, with a low absolute risk may be at a substantially relative higher has been addressed in the European guidelines [46], and this is particularly the case for HIV-1-infected patients because even if its absolute risk is low, it may still be 10-12 times higher than that of a person with low risk factor, usually because the addition of cumulative risk factors. The present study shows that the use of potent antiretroviral therapy resulting in more profound virus suppression and more preserved immunity, was associated with a high both relative and absolute risk of exhibiting risk factors for CHD. How these projections will translate to clinical events of CHD is difficult to predict, since it is assumed that there presumably will be a time lag from when factors known to accelerate the atherosclerotic process are induced and until clinical manifestations of atherosclerotic vascular disease occur. Present evidences suggest that HIV-1-infected patients have a 27% yearly risk of developing a cardiovascular event [47]. As many of these factors are likely to act synergistically, together with the underlying HIV-1 infection itself the time-lag cannot be reliably assessed. However, ongoing studies such as DAD [13] may provide fruitful comparisons between expected and observed CVD event rates [48]. Clearly, the management of CVR factors among our HIV-1-infected patients needs to be improved, and must follow two paths: a continued need for developing less harmful and better tolerated effective treatments for HIV-1 infection together with a better knowledge of pharmaceutical and non-pharmaceutical measures directed at reducing CVD risk by HIV-1 physicians. Given the high prevalence of smoking among our patients, strategies to quit smoking will likely prove cost-effective in preventing CVD in HIV-1-infected patients.

## JUSTIFICATION OF AUTHORSHIP

Pere Domingo, Ignacio Suárez-Lozano, and Myriam Garrido were in charge of the design, coordination and wrote the article. Pompeyo Viciana, Fernando Lozano, Alberto Terron, MJosé Galindo, Juan Gonzalez, Pere Domingo, Esteban Ribera, Antonio Vergara, Ramón Teira, Paloma Geijo, Jaime Cosín, Bernardino Roca, Belen de la Fuente, Trinitario Sanchez, contributed to de design of the computer application, data entry for the study, local coordination and bring up ideas to complete the final version of the manuscript. Juan R Lacalle and Ferran Torres made the statistic analyses.

## FINANCIAL SUPPORT

This work was possible through an unrestricted grant from Bristol, Myers &; Squibb.

## Figures and Tables

**Table 1:** Demographic, Laboratory, Clinical and Antiretroviral Therapy Characteristics of the VACH Population at Baseline Ac-cording to Current Usage of ART Drug Classes

**Current ART at Enrolment**	**NRTI Only (n = 233)**	**Combination with NNRTI (n = 1094)**	**Combination with PI (n = 990)**	**Combination with PI Plus NNRTI (n = 41)**	**Total (n = 2358)**	**p**
Age (years), median [IQR]^ &^	39.00 [35.00,43.00]	40.00 [35.00,45.00]	39.00 [36.00,43.00]	41.00 [38.00,44.00]	40.00 [35.00,44.00]	0.0852
Sex (% female) [95%CI]	54 (23.2%) [17.9%,29.1%]	243 (22.2%) [19.8%,24.8%]	217 (21.9%) [19.4%,24.6%]	8 (19.5%) [8.8%,34.9%]	522 (22.1%) [20.5%,23.9%]	0.9450
AIDS (%) [95%CI]	57 (24.5%) [19.1%,30.5%]	346 (31.6%) [28.9%,34.5%]	367 (37.1%) [34.1%,40.2%]	18 (43.9%) [28.5%,60.3%]	788 (33.4%) [31.5%,35.4%]	0.0005
** HIV Acquisition^&^**						< 0.0001
Injecting drug use	121 (52.2%) [45.5%,58.7%]	443 (40.8%) [37.9%,43.8%]	558 (56.7%) [53.5%,59.8%]	18 (43.9%) [28.5%,60.3%]	1140 (48.7%) [46.6%,50.7%]	
Homosexual	32 (13.8%) [9.6%,18.9%]	245 (22.6%) [20.1%,25.2%]	137 (13.9%) [11.8%,16.2%]	8 (19.5%) [8.8%,34.9%]	422 (18.0%) [16.5%,19.6%]	
Heterosexual	73 (31.5%) [25.5%,37.9%]	344 (31.7%) [28.9%,34.5%]	240 (24.4%) [21.7%,27.2%]	10 (24.4%) [12.4%,40.3%]	667 (28.5%) [26.6%,30.3%]	
Others	6 (2.6%) [1.0%,5.5%]	54 (5.0%) [3.8%,6.4%]	49 (5.0%) [3.7%,6.5%]	5 (12.2%) [4.1%,26.2%]	114 (4.9%) [4.0%,5.8%]	
CD4 cell count (x 106/l), median [IQR]^ &^	510.00 [358.00,735.00]	459.00 [288.00,688.00]	368.00 [219.50,568.50]	427.00 [251.00,756.00]	426.00 [256.00,651.00]	< 0.0001
HIV-1 RNA (log_10_), median [IQR]^ &^	1.69 [1.69,1.70]	1.69 [1.69,1.99]	1.70 [1.69,2.44]	1.70 [1.69,2.30]	1.69 [1.69,2.17]	< 0.0001
HIV-1 RNA copies/mL, median [IQR]^ &^	49.00 [49.00,50.00]	49.00 [49.00,97.00]	50.00 [49.00,275.00]	50.00 [49.00,200.00]	49.00 [49.00,148.00]	0.0010
HIV-1 RNA (log_10_), 0median [IQR]^ &^	1.69 [1.69,1.70]	1.69 [1.69,1.99]	1.70 [1.69,2.44]	1.70 [1.69,2.30]	1.69 [1.69,2.17]	< 0.0001
ART exposure [median (IQR)]	4.68 [2.88,6.27]	4.12 [2.01,6.01]	4.31 [1.73,6.22]	6.33 [4.32,8.94]	4.29 [1.98,6.19]	< 0.0001
HAART exposure [median (IQR)]	4.59 [2.71,5.96]	4.00 [1.93,5.61]	4.04 [1.64,5.80]	6.11 [4.10,6.95]	4.10 [1.90,5.79]	< 0.0001
Exposure to PI (%) [95%CI]	153 (65.7%) [59.2%,71.7%]	527 (48.2%) [45.2%,51.2%]	990 (100.0%) [99.7%,100.0%]	41 (100.0%) [93.0%,100.0%]	1711 (72.6%) [70.7%,74.4%]	< 0.0001
Duration (years), median [IQR]	4.27 [0.00,5.95]	0.00 [0.00,5.55]	3.80 [1.21,5.86]	6.11 [2.53,6.98]	3.26 [0.00,5.80]	< 0.0001
Exposure to NNRTIs (%) [95%CI]	88 (37.8%) [31.5%,44.3%]	1094 (100.0%) [99.7%,100.0%]	357 (36.1%) [33.1%,39.1%]	41 (100.0%) [93.0%,100.0%]	1580 (67.0%) [65.1%,68.9%]	< 0.0001
Duration (years), median [IQR]	0.00 [0.00,2.80]	2.69 [1.19,4.08]	0.00 [0.00,2.32]	3.16 [1.67,4.26]	1.51 [0.00,3.64]	< 0.0001
Exposure to NRTI (%) [95%CI]	233 (100.0%) [98.7%,100.0%]	1092 (99.8%) [99.3%,100.0%]	990 (100.0%) [99.7%,100.0%]	40 (97.6%) [87.1%,99.9%]	2355 (99.9%) [99.6%,100.0%]	0.0003
Duration (years), median [IQR]	4.68 [2.88,6.27]	4.10 [2.01,6.01]	4.31 [1.72,6.22]	6.33 [4.32,8.94]	4.28 [1.98,6.19]	< 0.0001

Descriptive statistics are n (%) [95%CI] for qualitative, and median [IQR] for qualitative variables. NRTI = nucleoside reverse transcriptase inhibitors, NNRTI = non-nucleoside reverse transcriptase inhibitors, PI = protease inhibitors, AIDS = acquired immunodeficiency syndrome, ART = antiretroviral therapy, HAART = highly active antiretroviral therapy. ^&^Percentage of missingness < 1%; null percentage for the rest of variables.

**Table 2 T2:** Cardiovascular Risk Factors in the VACH Cohort Population According to Current Usage of Antiretroviral Therapy (ART) Drug Classes

	** NRTI Only (n = 233)**	** Combination with NNRTI (n = 1094)**	** Combination with PI (n = 990)**	** Combination with PI Plus NNRTI (n = 41)**	** Total (n = 2358)**	**p**
Age > 45 years male, > 55 female, (%) [95%CI]^ &^	33 (14.2%) [10.0%,19.3%]	244 (22.3%) [19.9%,24.9%]	142 (14.4%) [12.2%,16.7%]	7 (17.1%) [7.2%,32.1%]	426 (18.1%) [16.5%,19.7%]	0.0001
Body mass index, median [IQR]	24.10 [22.28,26.22]	23.82 [21.81,25.59]	23.56 [21.52,25.56]	23.56 [21.05,25.95]	23.82 [21.75,25.67]	0.0700
Body mass index > 30 kg/m^2^, (%) [95%CI]	9 (3.9%) [1.8%,7.2%]	29 (2.7%) [1.8%,3.8%]	41 (4.1%) [3.0%,5.6%]	3 (7.3%) [1.5%,19.9%]	82 (3.5%) [2.8%,4.3%]	0.1428
Current smoker, (%) [95%CI]	146 (62.7%) [56.1%,68.9%]	588 (53.7%) [50.7%,56.7%]	641 (64.7%) [61.7%,67.7%]	22 (53.7%) [37.4%,69.3%]	1397 (59.2%) [57.2%,61.2%]	<0.0001
Family story of CVD, (%) [95%CI]	30 (12.9%) [8.9%,17.9%]	157 (14.4%) [12.3%,16.6%]	116 (11.7%) [9.8%,13.9%]	3 (7.3%) [1.5%,19.9%]	306 (13.0%) [11.6%,14.4%]	0.2227
Previous CVD, (%) [95%CI]	7 (3.0%) [1.2%,6.1%]	17 (1.6%) [0.9%,2.5%]	16 (1.6%) [0.9%,2.6%]	0 (0.0%) [0.0%,7.0%]	40 (1.7%) [1.2%,2.3%]	0.3520
Hypertension, (%) [95%CI]^ &^	50 (21.5%) [16.4%,27.3%]	272 (24.9%) [22.3%,27.5%]	212 (21.4%) [18.9%,24.1%]	5 (12.2%) [4.1%,26.2%]	539 (22.9%) [21.2%,24.6%]	0.0886
Diabetes mellitus, (%) [95%CI]	21 (9.0%) [5.7%,13.4%]	98 (9.0%) [7.3%,10.8%]	52 (5.3%) [3.9%,6.8%]	1 (2.4%) [0.1%,12.9%]	172 (7.3%) [6.3%,8.4%]	0.0046
Total cholesterol (mmol/l), median [IQR]	4.56 [3.83,5.13]	4.97 [4.20,5.78]	4.82 [4.12,5.65]	5.85 [4.61,6.94]	4.87 [4.12,5.67]	<0.0001
Total cholesterol > 6.2 mmol/l, (%) [95%CI]	11 (4.7%) [2.4%,8.3%]	172 (15.7%) [13.6%,18.0%]	145 (14.6%) [12.5%,17.0%]	18 (43.9%) [28.5%,60.3%]	346 (14.7%) [13.3%,16.2%]	<0.0001
HDL cholesterol (mmol/l), median [IQR]	1.09 [0.88,1.32]	1.27 [1.01,1.58]	1.17 [0.93,1.45]	1.19 [1.04,1.55]	1.19 [0.96,1.48]	<0.0001
HDL cholesterol < 0.9 mmol/l, (%) [95%CI]	60 (25.8%) [20.3%,31.9%]	157 (14.4%) [12.3%,16.6%]	216 (21.8%) [19.3%,24.5%]	6 (14.6%) [5.6%,29.2%]	439 (18.6%) [17.1%,20.2%]	<0.0001
TC/HDL ratio	4.20 [3.13,5.19]	3.87 [3.07,4.95]	4.17 [3.18,5.30]	4.72 [3.68,5.77]	4.05 [3.13,5.13]	<0.0001
LDL cholesterol (mmol/l), median [IQR]^ &^	2.51 [1.94,3.21]	2.90 [2.23,3.63]	2.75 [2.05,3.55]	3.44 [2.38,4.35]	2.81 [2.10,3.57]	<0.0001
LDL cholesterol > 4.14 mmol/l, (%) [95%CI]^ &^	11 (4.8%) [2.4%,8.4%]	142 (13.0%) [11.1%,15.2%]	122 (12.4%) [10.4%,14.6%]	12 (29.3%) [16.1%,45.5%]	287 (12.2%) [10.9%,13.6%]	<0.0001
Triglycerides (mmol/l), median [IQR]	4.56 [3.83,5.13]	4.97 [4.20,5.78]	4.82 [4.12,5.65]	5.85 [4.61,6.94]	4.87 [4.12,5.67]	<0.0001
Triglycerides > 2.3 mmol/l, (%) [95%CI]	58 (24.9%) [19.5%,31.0%]	239 (21.8%) [19.4%,24.4%]	294 (29.7%) [26.9%,32.7%]	20 (48.8%) [32.9%,64.9%]	611 (25.9%) [24.2%,27.7%]	<0.0001
Lipodystrophy, (%) [95%CI]	41 (17.6%) [12.9%,23.1%]	200 (18.3%) [16.0%,20.7%]	168 (17.0%) [14.7%,19.5%]	11 (26.8%) [14.2%,42.9%]	420 (17.8%) [16.3%,19.4%]	0.4027

Descriptive statistics are n (%) [95%CI] for qualitative, and median [IQR] for qualitative variables. NRTI = nucleoside reverse transcriptase inhibitors, NNRTI = non-nucleoside reverse transcriptase inhibitors, PI = protease inhibitors, CVD = cardiovascular disease, LDL = low density lipoprotein, HDL = high density lipoprotein, TC = total cholesterol. ^&^Percentage of missingness < 1%; null percentage for the rest of variables.

**Table 3:** Association of Current Antiretroviral Therapy (ART) with BMI, Hypertension, Diabetes, Dyslipidaemia, and Lipodystro-phy at Baseline. Results from Logistic Regression Models, Univariable and Adjusted for Other Factors

	**Only NRTI**	**NNRTI**				**PI**				**NNRTI+PI**				
	**Ref. **	**OR**	**95%CI**		**p**	**OR**	**95%CI**		**p**	**OR**	**95%CI**		**p**
**BMI>30kg/m**^2^													
Univariant	[1]	0.69	0.31	1.55	0.373	1.13	0.52	2.45	0.761	2.08	0.53	8.2	0.294
Multivariable	[1]	0.63	0.28	1.42	0.266	1.13	0.52	2.46	0.756	1.91	0.48	7.57	0.359
**Diabetes**													
Univariant	[1]	1.12	0.66	1.9	0.672	0.59	0.34	1.04	0.069	0.28	0.04	2.15	0.221
Multivariable	[1]	0.98	0.57	1.68	0.933	0.6	0.34	1.06	0.079	0.25	0.03	1.97	0.189
**HDL <;=0.9mmol/l **													
Univariant	[1]	0.48	0.34	0.68	0.000	0.79	0.56	1.11	0.174	0.49	0.19	1.22	0.124
Multivariable	[1]	0.45	0.32	0.65	0.000	0.67	0.47	0.95	0.025	0.38	0.15	0.98	0.045
**Hypolipemiant drugs**													
Univariant	[1]	1.26	0.52	3.02	0.61	0.76	0.3	1.92	0.566	NE	NE	NE	0.984
Multivariable	[1]	1.36	0.56	3.31	0.5	0.88	0.34	2.27	0.796	NE	NE	NE	0.983
**Hypertension**													
Univariant	[1]	1.35	0.94	1.94	0.105	1.1	0.76	1.59	0.599	0.57	0.21	1.53	0.265
Multivariable	[1]	1.28	0.88	1.88	0.198	1.26	0.86	1.86	0.238	0.57	0.21	1.59	0.283
**Lipodystrophy**													
Univariant	[1]	0.99	0.68	1.44	0.97	0.92	0.63	1.35	0.685	1.59	0.74	3.44	0.237
Multivariable	[1]	0.63	0.28	1.42	0.266	1.13	0.52	2.46	0.756	1.91	0.48	7.57	0.359
**Cardiovascular Risk (moderate-high)**													
Univariant	[1]	0.93	0.67	1.3	0.671	0.84	0.6	1.18	0.315	0.71	0.31	1.62	0.41
Multivariable	[1]	0.63	0.28	1.42	0.266	1.13	0.52	2.46	0.756	1.91	0.48	7.57	0.359
**Total Cholesterol >= 6.2 mmol/l**													
Univariant	[1]	3.54	1.89	6.64	0.000	3.21	1.7	6.04	0.000	14.8	6.23	35.14	0.000
Multivariable	[1]	3.34	1.77	6.31	0.000	4.04	2.12	7.71	0.000	17.77	7.24	43.59	0.000
**TG >= 2.3 mmol/l**													
Univariant	[1]	0.85	0.6	1.2	0.356	1.31	0.93	1.83	0.122	2.98	1.5	5.92	0.002
Multivariable	[1]	0.81	0.57	1.15	0.241	1.4	0.99	1.99	0.059	2.8	1.39	5.65	0.004

The final multivariable model included variables that were significantly (P<0.05) associated with the cardiovascular risk factor in question. The following variables were tested age, gender, smoking, family history of coronary heart disease, previous cardiovascular disease, body mass index, transmission mode, CD4 cell count, HIV RNA, previous AIDS. PI, protease inhibitor; NNRTI, non-nucleoside reverse transcriptase inhibitor; NRTI, nucleoside reverse transcriptase inhibitor; OR, odds ratio; CI, confidence interval; HDL, high den-sity lipoprotein; TG, triglycerides.

**Table 4:** Risk for BMI, Hypertension, Diabetes, Dyslipidaemia, and Lipodystrophy at Baseline. Per Year of ART Exposure for HAART, NNRTI, NRTI, and PI. Results from Logistic Regression Models, Univariable and Adjusted for Other Factors

	**Risk Per Year of HAA Exposure**				**Risk Per Year of NRTI Exposure**				**Risk Per Year of NNRTI Exposure**				**Risk Per Year of PI Exposure**			
	**OR**	**95%CI**		**p**	**OR**	**95%CI**		**p**	**OR**	**95%CI**		**p**	**OR**	**95%CI**		**p**
**BMI> 30kg/m^2^**																
Univariant	0.95	0.86	1.05	0.329	0.93	0.85	1.01	0.091	0.96	0.85	1.09	0.546	0.98	0.90	1.06	0.570
Multivariable	0.95	0.87	1.05	0.358	0.93	0.86	1.01	0.104	0.95	0.84	1.08	0.443	0.98	0.90	1.06	0.626
**Diabetes**																
Univariant	1.09	1.01	1.17	0.018	1.06	1.00	1.12	0.036	1.11	1.02	1.20	0.011	1.03	0.97	1.09	0.295
Multivariable	1.09	1.01	1.17	0.020	1.08	1.02	1.14	0.011	1.10	1.01	1.19	0.031	1.04	0.98	1.10	0.243
**HDL < =0.9 mmol/l**																
Univariant	0.99	0.94	1.03	0.590	1.00	0.96	1.03	0.834	0.93	0.88	0.99	0.018	1.01	0.97	1.05	0.627
Multivariable	1.01	0.96	1.06	0.638	1.01	0.97	1.05	0.633	0.96	0.91	1.02	0.221	1.02	0.98	1.06	0.391
**Hypolipemiant drugs**																
Univariant	1.18	1.06	1.32	0.003	1.11	1.02	1.20	0.014	1.22	1.08	1.39	0.002	1.08	0.99	1.18	0.097
Multivariable	1.16	1.03	1.30	0.014	1.10	1.01	1.20	0.032	1.19	1.05	1.36	0.007	1.06	0.97	1.16	0.209
**Hypertension**																
Univariant	1.06	1.02	1.11	0.004	1.05	1.02	1.09	0.003	1.07	1.02	1.13	0.007	1.03	1.00	1.07	0.070
Multivariable	1.06	1.01	1.11	0.017	1.06	1.02	1.10	0.002	1.06	1.00	1.11	0.051	1.03	1.00	1.07	0.070
**Lipodystrophy**
Univariant	1.32	1.25	1.39	0.000	1.19	1.15	1.24	0.000	1.16	1.10	1.23	0.000	1.21	1.16	1.26	0.000
Multivariable	1.30	1.23	1.37	0.000	1.18	1.14	1.23	0.000	1.13	1.07	1.20	0.000	1.19	1.14	1.24	0.000
**Risk (moderate-high)**																
Univariant	1.07	1.03	1.12	0.001	1.05	1.02	1.09	0.004	1.07	1.02	1.12	0.008	1.04	1.00	1.07	0.044
Multivariabl	1.07	1.02	1.12	0.004	1.07	1.03	1.11	0.001	1.05	0.99	1.11	0.079	1.04	1.00	1.08	0.041
**Total Cholesterol > = 6.2 mmol/l**																
Univariant	1.04	0.99	1.10	0.093	1.02	0.98	1.06	0.314	1.09	1.03	1.16	0.005	1.02	0.98	1.07	0.251
Multivariable	1.03	0.98	1.09	0.268	1.03	0.98	1.08	0.242	1.04	0.98	1.11	0.190	1.03	0.98	1.07	0.221
**TG > = 2.3 mmol/l**																
Univariant	1.06	1.02	1.11	0.003	1.05	1.02	1.09	0.001	1.02	0.97	1.07	0.370	1.06	1.02	1.09	0.001
Multivariable	1.04	1.00	1.09	0.068	1.04	1.01	1.08	0.016	0.99	0.94	1.04	0.770	1.05	1.01	1.08	0.012

The final multivariable model included variables that were significantly (P< 0.05) associated with the cardiovascular risk factor in question. The following variables were tested age, gender, smoking, family history of coronary heart disease, previous cardiovascular disease, body mass index, transmission mode, CD4 cell count, HIV RNA, previous AIDS. PI, protease inhibitor; NNRTI, non-nucleoside reverse transcriptase inhibitor; NRTI, nucleoside reverse transcriptase inhibitor; OR, odds ratio; CI, confidence interval; HDL, high den-sity lipoprotein; TG, triglycerides.

**Table 5:** Cardiovascular Risk Factors in the VACH Cohort Population According to the Presence of Fat Redistribution

** **	** Without LD (n=1938)**	** With LD (n=420)**	** Total (n=2358)**	**p**
Age (years), median [IQR]	39.00 [35.00,44.00]	41.00 [37.00,46.00]	40.00 [35.00,44.00]	<0.0001
Age > 45 years male, > 55 female, n (%) [95%CI]	331 (17.1%) [15.4%,18.8%]	95 (22.6%) [18.7%,26.9%]	426 (18.1%) [16.5%,19.7%]	0.0076
Body mass index, median [IQR]	23.80 [21.72,25.65]	23.83 [21.84,25.85]	23.82 [21.75,25.67]	0.5305
Body mass index > 30 kg/m2 median, n (%) [95%CI]	74 (3.8%) [3.0%,4.8%]	8 (1.9%) [0.8%,3.7%]	82 (3.5%) [2.8%,4.3%]	0.0523
Current smoker, n (%) [95%CI]	1141 (58.9%) [56.6%,61.1%]	256 (61.0%) [56.1%,65.6%]	1397 (59.2%) [57.2%,61.2%]	0.4322
Sex (female), n (%) [95%CI]	439 (22.7%) [20.8%,24.6%]	83 (19.8%) [16.1%,23.9%]	522 (22.1%) [20.5%,23.9%]	0.1959
Clinical AIDS, n (%) [95%CI]	630 (32.5%) [30.4%,34.6%]	158 (37.6%) [33.0%,42.4%]	788 (33.4%) [31.5%,35.4%]	0.0441
Clinical or Immunologic AIDS, n (%) [95%CI]	1100 (56.8%) [54.5%,59.0%]	268 (63.8%) [59.0%,68.4%]	1368 (58.0%) [56.0%,60.0%]	0.0080
CD4 cell count (x 106/l), median [IQR)]	414.00 [249.50,628.50]	480.00 [310.00,713.00]	426.00 [256.00,651.00]	<0.0001
Nadir CD4 cell count (x 106/l), median [IQR)]	141.00 [49.00,252.00]	130.00 [47.00,240.00]	140.00 [48.00,250.00]	0.3251
Time from HIV diagnosis (years), median [IQR)]	7.21 [4.02,12.47]	9.08 [6.26,13.98]	7.46 [4.42,12.75]	<0.0001
HIV-1 RNA (log10), median [IQR)]	1.70 [1.69,2.24]	1.69 [1.69,1.85]	1.69 [1.69,2.17]	0.0011
ART exposure (years), median [IQR)]	4.01 [1.72,5.95]	5.51 [3.75,6.85]	4.29 [1.98,6.19]	<0.0001
HAART exposure (years), median [IQR)]	3.80 [1.65,5.52]	5.29 [3.70,6.56]	4.10 [1.90,5.79]	<0.0001
Exposure to PI, n (%) [95%CI]	1363 (70.3%) [68.2%,72.4%]	348 (82.9%) [78.9%,86.3%]	1711 (72.6%) [70.7%,74.4%]	<0.0001
Duration (years), median [IQR)]	2.42 [0.00,5.53]	5.25 [1.98,6.64]	3.26 [0.00,5.80]	<0.0001
Exposure to NNRTIs, n (%) [95%CI]	1283 (66.2%) [64.0%,68.3%]	297 (70.7%) [66.1%,75.0%]	1580 (67.0%) [65.1%,68.9%]	0.0746
Duration (years), median [IQR)]	1.33 [0.00,3.45]	2.49 [0.00,4.17]	1.51 [0.00,3.64]	<0.0001
Exposure to NRTIs, n (%) [95%CI]	1935 (99.8%) [99.5%,100.0%]	420 (100.0%) [99.3%,100.0%]	2355 (99.9%) [99.6%,100.0%]	0.4198
Duration (years), median [IQR)]	4.00 [1.72,5.94]	5.51 [3.73,6.85]	4.28 [1.98,6.19]	<0.0001
Family story of CVD, n (%) [95%CI]	236 (12.2%) [10.8%,13.7%]	70 (16.7%) [13.2%,20.6%]	306 (13.0%) [11.6%,14.4%]	0.0131
Previous CVD, n (%) [95%CI]	33 (1.7%) [1.2%,2.4%]	7 (1.7%) [0.7%,3.4%]	40 (1.7%) [1.2%,2.3%]	0.9586
Hypertension, n (%) [95%CI]	412 (21.3%) [ 19.5%,23.2%]	127 (30.2%) [25.9%,34.9%]	539 (22.9%) [21.2%,24.6%]	<0.0001
Diabetes mellitus [% (95% CI)	121 (6.2%) [5.2%,7.4%]	51 (12.1%) [9.2%,15.7%]	172 (7.3%) [6.3%,8.4%]	<0.0001
Total cholesterol (mmol/l), median [IQR)]	4.87 [4.14,5.67]	4.84 [3.96,5.67]	4.87 [4.12,5.67]	0.2802
Total cholesterol > 6.2 mmol/l, n (%) [95%CI]	280 (14.4%) [12.9%,16.1%]	66 (15.7%) [12.4%,19.6%]	346 (14.7%) [13.3%,16.2%]	0.5061
HDL cholesterol (mmol/l), median [IQR)]	1.22 [0.98,1.50]	1.11 [0.91,1.40]	1.19 [0.96,1.48]	0.0002
HDL cholesterol < 0.9 mmol/l, n (%) [95%CI]	347 (17.9%) [ 16.2%,19.7%]	92 (21.9%) [18.0%,26.2%]	439 (18.6%) [17.1%,20.2%]	0.0562
TC/HDL ratio	4.00 [3.11,5.07]	4.24 [3.23,5.37]	4.05 [3.13,5.13]	0.0184
LDL cholesterol (mmol/l), median [IQR)]^ &^	2.82 [2.12,3.57]	2.77 [1.99,3.55]	2.81 [2.10,3.57]	0.3614
LDL cholesterol > 4.14 mmol/l, n (%) [95%CI]^ &^	235 (12.2%) [10.7%,13.7%]	52 (12.4%) [9.4%,16.0%]	287 (12.2%) [10.9%,13.6%]	0.8812
Triglycerides (mmol/l), median [IQR)]	1.53 [1.06,2.27]	1.70 [1.18,2.82]	1.55 [1.08,2.35]	<0.0001
Triglycerides > 2.3 mmol/l, n (%) [95%CI]	469 (24.2%) [22.3%,26.2%]	142 (33.8%) [29.3%,38.6%]	611 (25.9%) [24.2%,27.7%]	<0.0001
10-year cardiovascular risk estimate, n (%) [95%CI]				<0.0001
Low	193 (10.0%) [8.7%,11.4%]	68 (16.2%) [12.8%,20.1%]	261 (11.1%) [9.8%,12.4%]	
Moderate	1009 (52.1%) [49.8%,54.3%]	148 (35.2%) [30.7%,40.0%]	1157 (49.1%) [47.0%,51.1%]	
Moderate-high	495 (25.5%) [23.6%,27.5%]	145 (34.5%) [30.0%,39.3%]	640 (27.1%) [25.4%,29.0%]	
High	241 (12.4%) [11.0%,14.0%]	59 (14.0%) [10.9%,17.7%]	300 (12.7%) [11.4%,14.1%]	
Risk factor number, n (%) [95%CI]				<0.0001
0	542 (28.0%) [26.0%,30.0%]	71 (16.9%) [13.4%,20.8%]	613 (26.0%) [24.2%,27.8%]	
1	668 (34.5%) [32.4%,36.6%]	131 (31.2%) [26.8%,35.9%]	799 (33.9%) [32.0%,35.8%]	
≥ 2	728 (37.6%) [35.4%,39.8%]	218 (51.9%) [47.0%,56.8%]	946 (40.1%) [38.1%,42.1%]	

Descriptive statistics are n (%) [95%CI] for qualitative, and median [IQR] for qualitative variables. LD = lipodystrophy, AIDS = acquired immunodeficiency syndrome, ART = antiretroviral therapy, HAART = highly active antiretroviral therapy, CVD = cardiovascular disease, PI = protease inhibitor; NNRTI = non-nucleoside reverse transcriptase inhibitor; NRTI = nucleoside reverse transcriptase inhibitor; OR = odds ratio; CI = confidence interval; HDL= high density lipoprotein; TC = total cholesterol. ^&^Percentage of missingness <1%; null percentage for the rest of variables.

**Table 6:** Cardiovascular Risk Assessment and Need for Lipid-Lowering Therapy (LLT) in the VACH Cohort Population Accord-ing to Current Usage of Antiretroviral Therapy (ART) Drug Classes

	**NRTI Only**	**Combination with**	**Combination with**	**Combination with**	**Total**	**p**
	(n = 233)	NNRTI (n = 1094)	PI (n = 990)	PI plus NNRTI (n = 41)	(n = 2358)	
** Risk factor number, n (%) [95%CI]**						0.0001
0	42 (18.0%)	326 (29.8%)	228 (23.0%)	17 (41.5%)	613 (26.0%)	
	[13.3%,23.6%]	[27.1%,32.6%]	[20.4%,25.8%]	[26.3%,57.9%]	[24.2%,27.8%]	
1	78 (33.5%)	363 (33.2%)	345 (34.8%)	13 (31.7%)	799 (33.9%)	
	[27.4%,39.9%]	[30.4%,36.1%]	[31.9%,37.9%]	[18.1%,48.1%]	[32.0%,35.8%]	
≥ 2	113 (48.5%)	405 (37.0%)	417 (42.1%)	11 (26.8%)	946 (40.1%)	
	[41.9%,55.1%]	[34.2%,40.0%]	[39.0%,45.3%]	[14.2%,42.9%]	[38.1%,42.1%]	
**10-yr Cardiovascular risk estimate, n (%) [95%CI]**						
Low	107 (45.9%)	548 (50.1%)	475 (48.0%)	27 (65.9%)	1157 (49.1%)	0.0184
	[39.4%,52.6%]	[47.1%,53.1%]	[44.8%,51.1%]	[49.4%,79.9%]	[47.0%,51.1%]	
Moderate	65 (27.9%)	280 (25.6%)	289 (29.2%)	6 (14.6%)	640 (27.1%)	
	[22.2%,34.1%]	[23.0%,28.3%]	[26.4%,32.1%]	[5.6%,29.2%]	[25.4%,29.0%]	
Moderate-high	34 (14.6%)	124 (11.3%)	138 (13.9%)	4 (9.8%)	300 (12.7%)	
	[10.3%,19.8%]	[9.5%,13.4%]	[11.8%,16.3%]	[2.7%,23.1%]	[11.4%,14.1%]	
High	27 (11.6%)	142 (13.0%)	88 (8.9%)	4 (9.8%)	261 (11.1%)	
	[7.8%,16.4%]	[11.0%,15.1%]	[7.2%,10.8%]	[2.7%,23.1%]	[9.8%,12.4%]	
Lipid-lowering therapy, n (%) [95%CI]^ &^						
Needed	26 (11.3%)	201 (18.5%)	177 (17.9%)	11 (26.8%)	415 (17.7%)	0.0243
	[7.5%,16.1%]	[16.2%,20.9%]	[15.6%,20.5%]	[14.2%,42.9%]	[16.2%,19.3%]	
Current use	6 (2.6%)	36 (3.3%)	21 (2.1%)	0 (0.0%)	63 (2.7%)	0.2725
	[1.0%,5.5%]	[2.3%,4.5%]	[1.3%,3.2%]	[0.0%,7.0%]	[2.1%,3.4%]	
** Type of therapy, n (%) [95%CI]**						0.3161
None	227 (97.4%)	1058 (96.7%)	969 (97.9%)	41 (100.0%)	2295 (97.3%)	
	[94.5%,99.0%]	[95.5%,97.7%]	[96.8%,98.7%]	[93.0%,100.0%]	[96.6%,97.9%]	
Fibrates	3 (1.3%)	12 (1.1%)	12 (1.2%)	0 (0.0%)	27 (1.1%)	
	[0.3%,3.7%]	[0.6%,1.9%]	[0.6%,2.1%]	[0.0%,7.0%]	[0.8%,1.7%]	
Statines	3 (1.3%)	24 (2.2%)	9 (0.9%)	0 (0.0%)	36 (1.5%)	
	[0.3%,3.7%]	[1.4%,3.2%]	[0.4%,1.7%]	[0.0%,7.0%]	[1.1%,2.1%]	

Descriptive statistics are n (%) [95%CI] for qualitative, and median [IQR] for qualitative variables.^&^Percentage of missingness <1%; null percentage for the rest of variables.

## APPENDIX: CUTT-OFF VALUES FOR SPECIFICATION OF CVD RISK FACTORS IN HIV-1-INFECTED PATIENTS

The specification of risk factors is as follows. (i) Dyslipidemia: defined as elevated total cholesterol > 6.2 mmol/l (240 mg/dl), and/or decreased HDL-cholesterol <  0.9 mmol/l (35 mg/dl), and/or elevated triglycerides > 2.3 mmol/l (200 mg/dl). [The cut-offs applied are based on cut-offs for high risk for CVD in the NCEP guidelines.] (ii) Older age: age > 45 years for men and > 55 for women. (iii) Family history of coronary heart disease: first-degree relative with myocardial infarction before age 50. (iv) Previous CVD: patients’ own previous experience of myocardial infarction and/or stroke. (v) Hypertension: elevated systolic blood pressure > 140 mmHg and/or elevated diastolic blood pressure > 90 mmHg, or usage of anti-hypertensive drugs. (vi) Diabetes: history of diabetes or usage of anti-diabetic therapy. (vii) Body mass index (BMI) was stratified in four categories: underweight (BMI <  18 kg/m^2^), normal weight (BMI, 18–26 kg/m^2^), overweight (BMI, 26.1–30 kg/m^2^) and obesity (BMI,> 30 kg/m^2^). Obesity was considered a cardiovascular risk factor. (viii) Smoking: current cigarette smoking at inclusion in the study. (ix) Presence of clinical lipodystrophy was defined as either characteristic fat loss (from the face and/or extremities), central fat gain (abdominal and/or cervicodorsal) or mixed (at least one sign each of fat loss and central fat gain), as judged by the treating physician.

The specification of therapies to manage CVD risk factors were as follows: a) patients taking lipid-lowering agents b) anti-hypertensive medication c) anti-diabetic agents or insulin (derivatives) d) platelet aggregation inhibitors, and e) smoking cessation therapy.
